# Supplementary material for: Stability of Nine Time-Dependent Antibiotics for Outpatient Parenteral Antimicrobial Therapy (OPAT) Use
Source: Antibiotics (Basel). 2025 May 3;14(5):466. doi: 10.3390/antibiotics14050466 (PMC12108229; doi:10.3390/antibiotics14050466)
Supplement: Supplementary file 1 [file antibiotics-14-00466-s001.zip › antibiotics-3592201-supplementary.pdf]

SUPPLEMENTARY MATERIALS

Table S1: HPLC conditions for the antibiotic stability studies

Antibiotic	Mobile phase (v/v)	pH	Flow rate (mL/min)	Injection volume (μL)	Detection wavelength (nm)	Retention time (min)	Reference
Amoxicillin	Isocratic elution: 0.05 M NaH_2PO_4 buffer/methanol (95/5)	4.4	1.0	50	220	4.45	[22]
Cefepime	Isocratic elution: 0.005 M KH_2PO_4 buffer/methanol (90/10)	7.5	1.0	50	257	3.8	[23]
Cefiderocol	Gradient elution: 0.05 M KH_2PO_4 buffer and methanol	3.0	1.5	50	260	5.9	[24]
Cefotaxime	Gradient elution: phase A (0.05 M Na_2HPO_4 buffer/methanol (86/14)) and phase B (0.05 M Na_2HPO_4 buffer/methanol (60/40))	6.25	1.3	10	235	9.0	[25]
Cefoxitin	Isocratic elution: 0.005 M KH_2PO_4 buffer/methanol (80/20)	7.5	1.0	10	272	3.2	[23]
Ceftazidime	Isocratic elution: 0.1 M ammonium acetate/acetonitrile (90/10)	7.5	1.0	20	260	4.1	[26]
Piperacillin	Isocratic elution: 0.05 M KH_2PO_4 buffer/acetonitrile (55/45)	3.0	1.0	2	230	3.4	[27]
